# Are Fecal Metabolome and Microbiota Profiles Correlated with Autism Severity? A Cross-Sectional Study on ASD Preschoolers

**DOI:** 10.3390/metabo11100654

**Published:** 2021-09-26

**Authors:** Luca Laghi, Paola Mastromarino, Margherita Prosperi, Maria Aurora Morales, Sara Calderoni, Elisa Santocchi, Filippo Muratori, Letizia Guiducci

**Affiliations:** 1 Department of Agricultural and Food Sciences, University of Bologna, 47521 Cesena, Italy; l.laghi@unibo.it; 2 Section of Microbiology, Department of Public Health and Infectious Diseases, Sapienza University, 00185 Rome, Italy; paola.mastromarino@uniroma1.it; 3 Department of Developmental Neuroscience, IRCCS Fondazione Stella Maris, 56128 Pisa, Italy; sara.calderoni@fsm.unipi.it (S.C.); elisa.santocchi@fsm.unipi.it (E.S.); filippo.muratori@fsm.unipi.it (F.M.); 4 National Research Council, Institute of Clinical Physiology, 56124 Pisa, Italy; morales@ifc.cnr.it (M.A.M.); letiziag@ifc.cnr.it (L.G.); 5 Department of Clinical and Experimental Medicine, University of Pisa, 56124 Pisa, Italy

**Keywords:** autism spectrum disorders, gastrointestinal, microbiota, fecal metabolome, inflammation, metabolomics

## Abstract

Autism spectrum disorders (ASD) make up a heterogeneous group of neurodevelopmental disorders characterized by social and communication difficulties associated with repetitive and restrictive behaviors. Besides core features, metabolic imbalances, inflammation, gastrointestinal (GI) symptoms, and altered gut microbiota composition were often described in association with ASD, but their connection with the severity of autism (SA) remains unexplored. In this study, fecal metabolome, microbiota, and calprotectin levels of 80 ASD preschoolers were quantified and correlated with SA. Twelve of the fifty-nine molecules that were quantified by fecal metabolome analysis were significantly associated with SA. No links between SA or GI symptoms and microorganisms’ relative abundance were highlighted. Significant correlations between bifidobacteria, *Sutterella*, lactobacilli relative abundance, and metabolomics profiles were found. These results suggest that fecal metabolome discriminates the SA and intestinal microorganisms mediate the link between metabolome and SA regardless of GI symptomatology. The study raises the possibility that grouping ASD populations through metabolomics and fecal microbiota could aid the identification of specific ASD endophenotypes, on the basis of the SA. Mechanistic studies focusing on detected biomarkers might be an option for future studies.

## 1. Introduction

Autism spectrum disorders (ASD) are a clinically and genetically heterogeneous group of neurodevelopmental disorders characterized by socio-communicative difficulties as well as repetitive and restrictive behaviors [1]. Goodwin et al. [2] and subsequently other authors [3,4,5,6] noticed that a large proportion of ASD subjects has gastrointestinal (GI) dysfunctions, with diarrhea, constipation, and abdominal pain reported as the most common symptoms [7]. The underlying multifaceted mechanisms connecting ASD and GI problems have still not been completely elucidated, but several studies have focused on the gut microbiota as a potential link between ASD and GI symptoms.

Indeed, some microbiota perturbations have been consistently found in ASD subjects, suggesting the possible role of the gut microbiota as a contributing factor in the etiopathogenesis of ASD [8]. Several studies suggested that decreases of *Bifidobacterium* spp. and increases of *Bacteroides* spp. in stool samples characterize the microbiota of ASD individuals [9]. An increase in *Prevotella* spp., belonging to the same phylum *Bacteroidetes*, has also been frequently detected. Moreover, variations of different clostridial clusters, *Sutterella* and *Akkermansia* genera gave contradictory results across studies.

The advent of techniques for the bulk measurement of genome, transcriptome, proteome, and metabolome has offered means to study microbial community’s overall traits. In this framework, the metabolome is considered the most convenient representation because minimal perturbations of the microbial profile may strongly impact molecules’ concentration, through cascade phenomena and pleiotropic effects [10]. For this reason, fecal metabolome has often been considered as a link between microbiota and host, especially concerning gastrointestinal symptoms [11,12]. Moreover, the concentration of some molecules in feces has been found to differentiate ASD subjects and typical counterparts. For instance, Kang [13] found that isopropanol and p-cresol concentrations were significantly higher in the feces of children with ASD than in controls, while gamma-aminobutyric acid showed an opposite trend. High concentrations of glutamate [14] and short-chain fatty acids [15] have also been detected in ASD children.

Interestingly, the totality of the studies focusing on the fecal metabolome features in ASD has investigated the differences between subjects with and without this disorder, while ignoring potential correlations between metabolome and ASD severity. The present work aimed at identifying possible correlations between water-soluble fecal metabolome, fecal microbiota, calprotectin levels (a biomarker of intestinal inflammation), and ASD severity in a group of ASD preschoolers with or without GI symptoms.

## 2. Results

### 2.1. Fecal Metabolome

Links with gastrointestinal disease.Fecal metabolome analysis allowed the identification and quantification of 59 molecules. To evaluate the metabolome’s features related to gastrointestinal symptoms, we applied a *t*-test on each molecule’s concentration and found that twelve of them significantly differed between children with and without gastrointestinal symptoms (GI and NGI children, respectively; Table 1a and Table S1).

Low vs. high autism severity autism diagnostic observation schedule (ADOS) scores. To evaluate the metabolome’s features potentially related to autism independently from the presence of gastrointestinal symptoms, we focused on children classified as low-ADOS and high-ADOS, and we applied a two-way ANOVA (gastrointestinal symptoms—ADOS) on each molecule. A preliminary investigation by Spearman correlation allowed us to observe that the specific samples considering ADOS symptoms were independent of GI symptomatology, measured as a GI score (*p* = 0.60, *r* = 0.06). We found twelve molecules that were significantly affected by ADOS (Table 1b and Table S2).

**Figure 1:**
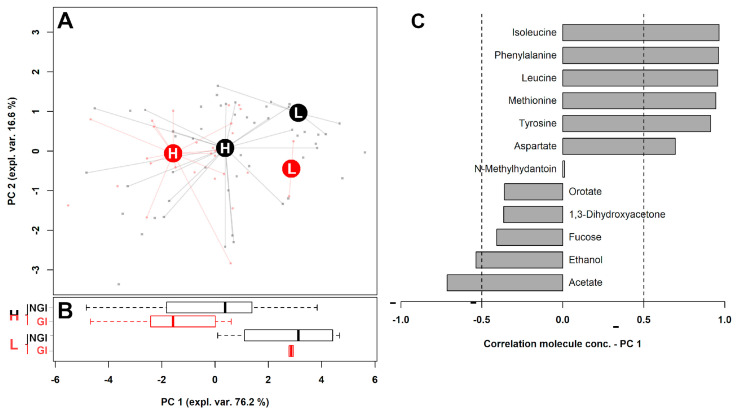
rPCA model calculated on fecal samples metabolomics of molecules reported in Table 1b distinguishing children within low-ADOS (L) and high-ADOS (H) groups. (**A**) The score plot reports the samples joined with lines to their median values, represented with big filled circles. Children without (NGI) and with gastrointestinal symptoms (GI)are evidenced with black or red colors, respectively. (**B**) Boxplot summarizing the position of the groups along with PC 1. (**C**) Correlation plot reporting the correlation between each substance’s importance over PC 1 and its concentration. Gray bars highlight significant correlations (*p* < 0.05).

To observe the overall distribution of the samples from low-ADOS and high-ADOS children in the space constituted by this reduced group of metabolites, we performed a rPCA model calculation distinguishing GI and NGI children (Figure 1A). Moreover, we performed a Boxplot representation summarizing the position of these groups along with PC 1 (Figure 1B), and a correlation plot reporting the correlation between each substance’s importance over PC 1 and its concentration (Figure 1C).

Three principal components (PCs) were included (described in detail in Table S3), the first of which accounted for 76.2% of the samples’ variance overall represented by the model. Along this PC, the samples appeared as distributed according to ADOS, with low-ADOS and high-ADOS children appearing at positive and negative PC 1 scores, respectively. To check the authenticity of the differences highlighted between high and low ADOS subjects, patients with intermediate ADOS were employed as a test set, by excluding them from any calculation and by predicting them through their projection over the PCA space based on the other subjects.

**Figure 2:**
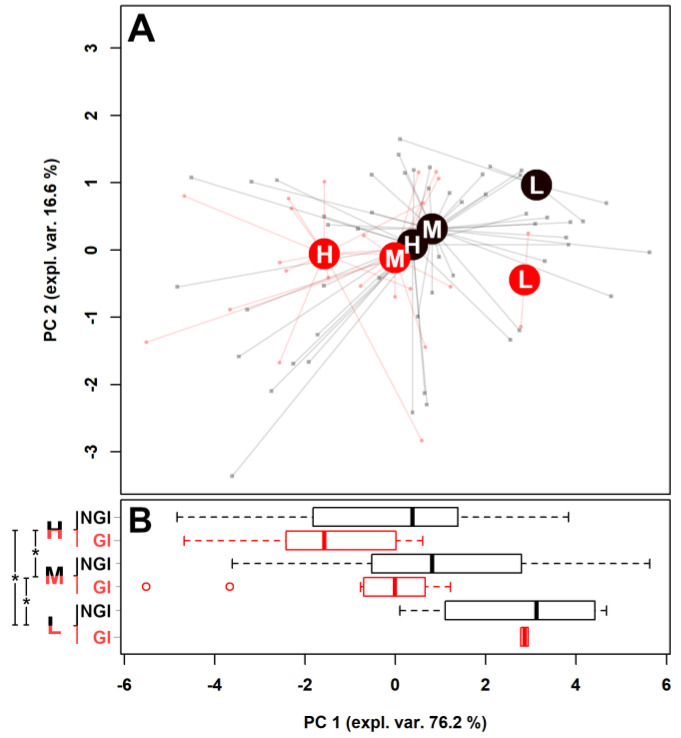
rPCA model calculated on the fecal metabolomics related with the molecules reported in Table 1b and adding the values relative to moderate-ADOS group (Table 2). (**A**) rPCA model of Figure 1 where moderate-ADOS children (M) were added within low-ADOS (L) and high-ADOS (H) groups. The score plot reports the samples joined with lines to their median values, represented with big filled circles. Children without (NGI) and with gastrointestinal symptoms (GI) are evidenced with black or red colors, respectively. (**B**) Boxplot summarizing the position of the groups along PC 1. Asterisks denote significant (*p* < 0.05) pairwise differences. Children without (NGI) and with gastrointestinal symptoms (GI) are evidenced with black or red colors, respectively.

Moderate autism severity. The molecules significantly which were different between low-ADOS and high-ADOS children were also quantified in the feces of moderate-ADOS children (Table 2), which were then projected over the rPCA space calculated before (Figure 2).

Both moderate-ADOS + NGI and moderate-ADOS + GI children appeared as characterized by PC 1 scores intermediate to the corresponding low-ADOS and high-ADOS. In addition, the presence of gastrointestinal symptoms separated NGI and GI children by 17.39%, a value that is intermediate between the values of 6.34% and 41.31% observed above.

**Figure 3:**
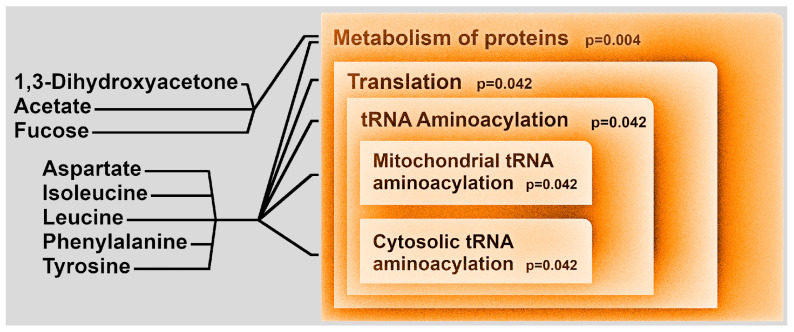
Metabolic pathways over-representation analysis based on the molecules significantly differed between low-ADOS and high-ADOS children. Orange colors are used for pathways significantly over-represented. Pathways are sub-leveled following Reactome’s hierarchy.

To obtain from the 12 molecules hints about the metabolic pathways most likely related to autism severity, we set up an overrepresentation analysis. Eight of them were found to be involved in the metabolism of proteins: 1,3-dihydroxyacetone, acetate, fucose, aspartate, isoleucine, leucine, phenylalanine, and tyrosine. Among them, the presence of five amino acids suggested that the alteration of the metabolism of proteins could be linked to their synthesis (Figure 3).

### 2.2. Fecal Microbiota and Intestinal Inflammation

The absolute abundance of total bacteria, lactobacilli, bifidobacteria, *Akkermansia muciniphila*, *Bacteroides*, *Prevotella*, and *Sutterella* are reported in Table S4, while Table S5 reports the relative abundance of the same bacterial groups. These microorganisms were chosen because several publications reported significant changes in their fecal levels in autistic subjects compared to healthy individuals [9]. The amount of total bacteria was similar between NGI and GI subjects, while the relative abundance of bifidobacteria was significantly lower in GI than in NGI children (*p* = 0.032). Moreover, although not significant, the relative abundance of *Prevotella* and *Sutterella* were higher in GI subjects, while the relative abundance of *A. muciniphila* was lower in the same subjects.

Intestinal inflammation in GI and NGI children was evaluated by fecal calprotectin. The median concentration of fecal calprotectin (Table S6) was not significantly different between NGI (79.27 μg/g, with a IQR of 131.15) and GI groups (69.50 μg/g, with a IQR of 131.21). Besides, no significant difference was observed between NGI and GI patients in the amount of calprotectin even when age-based stratification of children was considered.

In adults and children over 4 years old, values of fecal calprotectin below 50 μg/mg are generally viewed as normal. Intermediate levels (in the 50 and 200 μg/mg range) are considered to indicate low-grade intestinal inflammation, while values above 200 μg/mg are viewed as associated with pathology above [16]. The relative amount of *Akkermansia muciniphila* showed a negative correlation (*p* = 0.041, *r* = −0.32) with intermediate fecal calprotectin levels (50–200 μg/g). This correlation was stronger (*p* = 0.0002, *r* = −0.49) when all values of calprotectin higher than 50 μg/g were considered. On the contrary, *Prevotella* was directly correlated with levels of calprotectin higher than 200 μg/g (*p* = 0.0030, *r* = 0.75).

*Correlation among microorganisms*. As reported in Table S7, a positive linear correlation between the relative abundance of lactobacilli and bifidobacteria was observed in the entire study group (*p* = 0.0008, *r* = 0.38). Positive correlations were also observed between lactobacilli and *Prevotella* and between *Bacteroides* and *Sutterella*. Negative correlations were observed across the entire study group for bifidobacteria with *Bacteroides*, for lactobacilli with *Sutterella*, and for *Akkermansia* with *Bacteroides*. Finally, a negative correlation of bifidobacteria with *Sutterella* was consistently present in the entire study group and in NGI and GI groups observed separately.

*Low vs. High ADOS*. We first focused on children of the low-ADOS and high-ADOS groups. A two-way ANOVA (ADOS—GI) showed (Table S8) that ADOS was not significantly associated with any bacterial groups. Focusing on gastrointestinal problems, *Sutterella* showed a relative abundance significantly higher in GI subjects (*p* = 0.036), while *Prevotella* showed an opposite trend (*p* = 0.056).

We searched for microorganisms that could have determined the trends of the samples highlighted by the rPCA model of Figure 1. We found that the relative abundance of bifidobacteria (*r* = 0.38, *p* = 0.021) and *Sutterella* (*r* = −0.48, *p* = 0.0029) significantly correlated with samples’ scores along PC 1.

*Extension to the moderate ADOS group*. When the observation was extended to include moderate group, no links between ADOS or gastrointestinal disease and microorganisms’ relative abundance could be highlighted. For completeness, the absence of correlation was confirmed when correlations were calculated between microorganism’s abundance and ADOS score. In contrast, as highlighted in Table 3, when considering all the subjects studied, the significant correlation between bifidobacteria (*r* = 0.46) and *Sutterella* (*r* = −0.35) relative abundance and PC 1 was confirmed, and one with lactobacilli could be noticed (*r* = 0.27, *p* = 1.96 × 10^−2^).

## 3. Discussion

This study aims to identify whether water-soluble fecal metabolome, microbiota, and calprotectin levels correlate with SA levels in a group of ASD preschoolers. The metabolomic analysis highlighted a close relationship between the ASD severity and the fecal metabolic profile. Specifically, the 12 molecules differentiating low-ADOS and high-ADOS children can pave the way for further progress into the possible role of fecal metabolomics as a biomarker in autism.

A previous investigation demonstrated that the urine metabolome of young autistic children correlates with their clinical profile severity [17]. In our study, the six molecules significantly higher in the low-ADOS group than in the high-ADOS group were all amino acids, whereas none of the six molecules higher in the High-ADOS group was an amino acid. So, these metabolites seem to be able to distinguish children with severely impaired behaviors from those with lower severity of autism symptoms. De Angelis et al. [18] have found that proteolytic bacteria (e.g., *Clostridium* and *Bacteroides*) hydrolyzed proteins and peptides, producing consistent amounts of free amino acids (FAA) detectable in the fecal samples of ASD children. In a previous study, the same authors [14] reported higher levels of aspartate, an excitatory neurotransmitter acting on *N*-methyl-d-aspartate (NMDA) receptors in the feces of children with ASD, compared to typically developing peers. Kang and colleagues [13] also observed relatively higher concentrations in their ASD group, which may reflect its potential contribution to ASD symptoms associated with *N*-methyl-d-aspartate (NMDA) receptor dysfunction. In fact, dysfunctional ionotropic NMDA receptors have been recently linked to multiple forms of ASD and emerging evidence showed that d-aspartate and d-serine are important neuromodulators of glutamatergic transmission [19]. These data suggest that targeting the NMDA receptor could have promising therapeutic potential in ASDs and experimental studies have been conducted with this aim [20].

The lack of other studies investigating the correlations between autism severity and amino acids concentrations prevents us from further speculation besides the significant correlation between fecal aspartate levels and ADOS.

Among the fecal non-amino acidic molecules, which significantly vary between children with high and low ASD severity, three molecules (fucose, 1,3-dihydroxyacetone, *N*-methylhydantoin) are of particular interest since they characterized the highest severity group, both with and without GI symptoms.

As far as we know, the fucose’s role has never been studied in subjects with ASD to date. Considering that synaptic plasticity, neurite outgrowth and neuron morphology are regulated by fucosylation and are responsible for several cognitive processes, including learning and memory, this molecule deserves further investigation [21]. The fucose is released in the colon by commensal intestinal bacteria (from some strains of Bifidobacteria) that cleave fucose residue from the chain of glycolipid and utilize it as the carbon source [22]. Therefore, since fucose is metabolized by various bacterial strains, the different fucose concentrations in our fecal samples could be justified by a different gut microbial composition. Moreover, it has been shown that fucose has an anti-inflammatory role against intestinal infections [23], modulating the interaction between gut microbiota and bile acid in animal models [22].

Concerning the dihydroxyacetone, it contributes to the oxidative phosphorylation pathway in the mitochondria to generate ATP. Therefore, the alteration of dihydroxyacetone levels in ASD, and in the High-ADOS group, in particular, could be ascribed to the hypothesis that mitochondrial dysfunction is associated in a subset of subjects with ASD [24]. The role of the *N*-methylhydantoin remains even more enigmatic and unexplored in autism; it is a bacterial metabolite, i.e., the product of degradation of creatinine by bacteria [25]. To the best of our knowledge, it has never been studied in autism, and further investigations are needed to clarify its role.

Moreover, our results indicate that the above-mentioned metabolites discriminate between children with severely impaired behaviors and low impaired behaviors. In fact, the mild-moderate profile is positioned in the middle, indicating a continuous trend between the severity of ASD and the fecal concentration of these molecules. Thus, to the best of our knowledge, the current study represents the first attempt to identify a fecal metabolomic cluster distinguishing different levels of autism severity.

Metabolic pathways over-representation analysis based on the molecules that significantly differentiate between low-ADOS and high-ADOS children showed the involvement of proteins’ metabolism, particularly the tRNA aminoacylation, both at the mitochondrial and cytosolic level. Aminoacyl-tRNA synthetases (ARSs) are a ubiquitously expressed family of nuclear enzymes responsible for charging tRNAs with their relative amino acids, therefore fundamental for the first step in protein synthesis. The role of tRNA synthetases has been studied in neurological and neuromuscular disorders [26] and changes in protein synthesis have been previously observed in mouse models of ASD/intellectual disability [27,28]. In fact, the de novo protein synthesis plays a pivotal role in regulating the synaptic function and plasticity; mutations in several genes involved in the regulation of protein synthesis have been identified as risk factors for the development of ASD with associated intellectual disability [29].

Therefore, we could conclude that the over-representation analysis of the molecules significantly differing between low-ADOS and high-ADOS children highlighted a metabolic pathway that might potentially be involved in ASD.

Besides metabolomics analysis, the microbiota characterization that we performed indicated that microorganisms and inflammation mediate the link between metabolome and severity of autism.

The recent literature detected a different microbial composition between ASD children and typically developing controls [30,31], suggesting the presence of dysbiosis in ASD children [32]. De Angelis et al. [14] studied children with pervasive developmental disorder (not otherwise specified) and Autistic Disorder (AD) in comparison to typically developing controls, demonstrating a higher deviation of relative abundance of fecal microbiota in the AD group compared to the other two groups.

Our results showed no difference in the absolute abundance of total bacteria between NGI and GI subjects. Conversely, the relative abundance of bifidobacteria was significantly lower in GI than in NGI children. Other studies investigating the gut microbial composition in ASD with or without GI symptomatology concluded that autism-related changes in both overall diversity and individual *genus* abundances were correlated with the presence of autistic symptoms, but not with their diet patterns [33]. In fact, notwithstanding it is well known that children with ASD are at higher risk of GI symptoms [34], few studies have characterized the different microbial profiles between ASD subjects with and without GI symptoms and considered them in the statistical analyses [35,36,37,38,39]. Our results provide evidence of altered gut microbiota in ASD children with GI symptoms, adding to the complexity of microbial differences in autism. The significantly lower relative levels of bifidobacteria in ASD children with GI symptoms than in those without GI symptoms confirm previous results of protective effects of these microbes for the gut and for human health in general [40]. However, the current findings do not allow us to establish whether these bacteria are implicated as a cause or a consequence of some GI symptoms.

In our results, the fecal calprotectin concentration was elevated and not significantly different in subjects with and without gastrointestinal symptoms. The observed values were similar to those reported by Zhu et al. [41] in typically developing Chinese children of similar ages. Conversely, the elevated concentration of calprotectin was directly correlated with *Prevotella* and inversely correlated with *Akkermansia*, suggesting an inflammatory and protective role of these two groups of microorganisms, respectively. In this context, some species of *Akkermansia* have been specifically observed in children with ASD [14,33], but their pathogenic role remains to be established.

To the best of our knowledge, we are among the few authors to have tested the link between the severity of autistic symptoms and the microbiota composition [42], highlighting the association between the SA and microbiota in parallel with the metabolomics analysis. By examining the severity of the autistic symptomatology, we did not observe differences in bacterial levels between Low-ADOS and High-ADOS groups. On the other hand, searching for microorganisms that could have determined the trends of the molecules highlighted by the rPCA model identifying low- and high-ADOS groups, we found that the relative abundance of Bifidobacteria and *Sutterella* significantly correlated with samples’ scores along with PC 1. This result suggests a link between microbial status and characteristics of metabolome profile in the two groups with different ASD severity. When considering all the subjects, the significant correlation between the relative abundance of bifidobacteria and *Sutterella* and PC 1 was confirmed (*r* = 0.46 and −0.35, respectively).

As mentioned above, bifidobacteria and lactobacilli, i.e., important components of the human gut microbiome, have health-promoting properties and contribute to the host homeostasis [40,43]. Crucially, *Bifidobacterium* and *Lactobacillus* levels positively correlated with amino acids and negatively correlated with the molecules characterizing the high-ADOS groups.

This last result allows us to hypothesize a link between fecal microbiota composition and water-soluble fecal metabolome as well as the higher relative abundance of *Lactobacillus* and *Bifidobacterium*, which is indicative of a metabolomic profile characterized by a lower ASD severity degree. Concerning *Sutterella*, other authors have previously reported high levels of these bacteria in the stools of children with ASD compared to healthy controls [36] as well as high rates of these bacteria in GI biopsies taken from ASD children with GI symptoms [44]. The consequences of an increase in *Sutterella* in the fecal populations are not yet known; however. it is possible that under certain conditions, these bacteria can cause infections [44].

We acknowledge the following limitations of this study. Firstly, we did not consider the dietary patterns of the enrolled subjects, which may have influenced their fecal microbiota, especially considering the frequent presence of food selectivity in this population. Secondly, we did not consider other members of gut microbial communities in addition to the investigated bacterial taxa as potential contributors to the metabolite profiles. Finally, the sample size of the low-ADOS group was smaller than the ones of the other groups.

In conclusion, our study represents the first to detect a direct correlation between degrees of autism severity, as well as the features of the water-soluble fecal metabolome, fecal microbiota, and calprotectin levels in a group of ASD preschoolers. These results pave the way for subtyping ASD population through the identification of specific metabolomic endophenotypes with the final aim of contributing to personalized therapies, given the need for evidence for personalized biopsychosocial interventions with this population.

## 4. Materials and Methods

### 4.1. Subjects

A total of 80 ASD preschoolers recruited from November 2015 to February 2018 during a clinical trial on the efficacy of probiotic supplementation in ASD preschoolers [45,46] were included in this study. The clinical data and the fecal samples were collected at baseline (Table 4).

### 4.2. Clinical Assessments

A multidisciplinary team performed the ASD diagnosis according to the Diagnostic and Statistical Manual of Mental Disorders (DSM)-5 [1] criteria and confirmed by Autism Diagnostic Observation Schedule-2 (ADOS-2) [47], a semi-structured assessment considered as the gold standard for the diagnosis of ASD with a demonstrated inter-rater reliability, test-retest reliability, and internal validity. Detailed information on the study design, inclusion, and exclusion criteria were reported in the study protocol [45].

All children had a comprehensive evaluation including anthropometric measures. The ADOS-calibrated severity score (ADOS-CSS) [48,49] was used to standardize and compare ADOS-2 raw scores across different modules and ages. The participants were divided into three subgroups characterized by low, moderate, and high ADOS-CSS scores, according to validated cut-offs for these three categories (low severity 1–4; moderate severity 5–7; high severity 8–10).

A modified version of the GI severity index (GSI [50]) allowed the detection of GI symptoms, splitting the subjects into two groups (GI vs. No-GI). A total score of 4 and above (with at least 3 score points from the first six items) was considered clinically significant for the classification of a subject within the GI group. Characteristics of the recruited subjects in the whole sample and in the three severity subgroups are reported in Table 4.

### 4.3. Metabolomics Analysis by ^1^H-NMR

Fecal metabolome was characterized and quantified by ^1^H-NMR, an analytical platform that grants a high reproducibility and requires a minimal sample preparation (see Appendix A (Appendix A.1) for further information).

### 4.4. Microbiota Analysis

DNA was extracted from fecal samples stored at −80 °C by QIAamp PowerFecal DNA Kit (Qiagen, Hilden, Germany). According to the manufacturer’s instructions, each sample was homogenized in a 2-mL bead beating tube containing garnet beads. DNA was eluted with Solution C6 and stored at −20 °C for further real-time qPCR analysis.

The quantification of total bacteria was performed using a universal primer set specific for 16S rDNA of domain bacteria and conditions, as reported elsewhere [51]. See Appendix A (Appendix A.2) for further information.

### 4.5. Calprotectin Analysis

According to the manufacturer’s instructions (BÜHLMANN fCAL^®^ ELISA, Buhlmann, Switzerland), fecal calprotectin levels were determined by means of a commercially available Enzyme-Linked Immunosorbent Assay (ELISA).

### 4.6. Statistical Analysis

Statistical analysis was conducted in an R computational language [52], while artwork was refined by GIMP (version 2.10, www.gimp.org (accessed on 12 September 2021)). Comparisons were performed by *t*-test or two-way ANOVA with interaction on data checked for normality and normalized when needed according to Box and Cox [53]. Correlations were investigated according to Spearman. A *p*-value of 0.05 was accepted as a limit for significance.

Trends underlying groups of molecules were highlighted by robust principal component analysis (rPCA) models [54], by employing the PcaHubert algorithm implemented in the rrcov package. In the first stage, the algorithm detects outlying samples according to their distance from the others along and orthogonally to the PCA plane. In the second stage, the optimal number of principal components (PCs) are determined. A scoreplot and a correlation plot summarize the main features of a rPCA model. The former represents the samples in the PC space, therefore evidencing the overall structure of the data. The latter report the correlations between the concentration of each variable and the model’s components, thus showing which molecule mostly determines the structure of the data.

Metabolic pathways over-representation analysis was performed by Fisher exact test, by employing Reactome (https://reactome.org (accessed on 12 September 2021)) as a pathways’ database.

## Figures and Tables

**Table 1:** Concentration (mmol/L; median (IQR)) in feces of the molecules significantly different between children (**a**) without (NGI) and with gastrointestinal symptoms (GI); (**b**) with high and low autism diagnostic observation schedule (ADOS) scores. Legend: ↑ higher;↓ lower.

(**a**)				
	**NGI**	**GI**	***p*-Value**	**GI vs. NGI**
Acetate	6.32 × 10^−2^ (4.06 × 10^−2^)	7.58 × 10^−2^ (4.99 × 10^−2^)	0.042	↑
Alanine	6.12 × 10^−3^ (2.10 × 10^−3^)	4.80 × 10^−3^ (1.62 × 10^−3^)	0.012	↓
Ethanol	4.62 × 10^−4^ (8.20 × 10^−4^)	5.78 × 10^−4^ (9.06 × 10^−4^)	0.048	↓
Formate	1.20 × 10^−4^ (5.59 × 10^−5^)	1.42 × 10^−4^ (9.45 × 10^−5^)	0.006	↑
Isoleucine	1.68 × 10^−3^ (8.23 × 10^−4^)	1.43 × 10^−3^ (5.31 × 10^−4^)	0.039	↓
Leucine	4.34 × 10^−3^ (2.31 × 10^−3^)	3.61 × 10^−3^ (1.16 × 10^−3^)	0.034	↓
Methionine	9.03 × 10^−4^ (3.75 × 10^−4^)	7.87 × 10^−4^ (3.02 × 10^−4^)	0.014	↓
Orotate	5.89 × 10^−5^ (4.18 × 10^−5^)	7.70 × 10^−5^ (6.42 × 10^−5^)	0.015	↑
Phenylalanine	1.34 × 10^−3^ (6.20 × 10^−4^)	1.19 × 10^−3^ (4.83 × 10^−4^)	0.037	↓
Propionate	1.82 × 10^−2^ (1.20 × 10^−2^)	2.23 × 10^−2^ (1.30 × 10^−2^)	0.035	↑
Tyrosine	2.76 × 10^−3^ (1.16 × 10^−3^)	2.47 × 10^−3^ (1.02 × 10^−3^)	0.048	↓
Uridine	4.55 × 10^−5^ (3.82 × 10^−5^)	6.51 × 10^−5^ (5.33 × 10^−5^)	0.003	↑
(**b**)				
	**Low-ADOS**	**High-ADOS**	***p*-Value** **Low vs. High-ADOS**	
1,3-Dihydroxyacetone	8.08 × 10^−5^ (6.53 × 10^−5^)	1.67 × 10^−4^ (1.65 × 10^−4^)	0.037	
Acetate	4.17 × 10^−2^ (1.14 × 10^−2^)	7.87 × 10^−2^ (3.72 × 10^−2^)	0.011	
Aspartate	2.16 × 10^−3^ (4.78 × 10^−4^)	1.18 × 10^−3^ (6.75 × 10^−4^)	1.78 × 10^−4^	
Ethanol	1.97 × 10^−4^ (2.91 × 10^−4^)	1.02 × 10^−3^ (1.10 × 10^−3^)	0.007	
Fucose	7.40 × 10^−5^ (3.47 × 10^−5^)	1.37 × 10^−4^ (8.47 × 10^−5^)	0.021	
Isoleucine	2.15 × 10^−3^ (4.07 × 10^−4^)	1.34 × 10^−3^ (7.11 × 10^−4^)	0.006	
Leucine	5.40 × 10^−3^ (9.46 × 10^−4^)	3.39 × 10^−3^ (2.04 × 10^−3^)	0.007	
Methionine	1.19 × 10^−3^ (2.78 × 10^−4^)	8.04 × 10^−4^ (3.35 × 10^−4^)	0.004	
*N*-Methylhydantoin	1.89 × 10^−5^ (8.73 × 10^−6^)	3.93 × 10^−5^ (4.35 × 10^−5^)	0.026	
Orotate	3.12 × 10^−5^ (1.55 × 10^−5^)	6.34 × 10^−5^ (6.89 × 10^−5^)	0.011	
Phenylalanine	1.57 × 10^−3^ (2.81 × 10^−4^)	1.16 × 10^−3^ (6.09 × 10^−4^)	0.005	
Tyrosine	3.24 × 10^−3^ (6.49 × 10^−4^)	2.42 × 10^−3^ (1.13 × 10^−3^)	0.011	

**Table 2:** Concentration (mmol/L; median (IQR)) in the samples from moderate-ADOS children of the molecules significantly different between samples from high-ADOS and low-ADOS children.

	NGI	GI	*p* Value
1,3-Dihydroxyacetone	1.50 × 10^−4^ (1.51 × 10^−4^)	2.59 × 10^−4^ (1.90 × 10^−4^)	NS
Acetate	6.52 × 10^−2^ (3.96 × 10^−2^)	5.49 × 10^−2^ (2.03 × 10^−2^)	NS
Aspartate	1.26 × 10^−3^ (6.79 × 10^−4^)	1.22 × 10^−3^ (6.84 × 10^−4^)	NS
Ethanol	3.59 × 10^−4^ (3.45 × 10^−4^)	5.42 × 10^−4^ (9.63 × 10^−4^)	NS
Fucose	1.01 × 10^−4^ (7.41 × 10^−5^)	1.15 × 10^−4^ (4.43 × 10^−5^)	NS
Isoleucine	1.68 × 10^−3^ (9.22 × 10^−4^)	1.55 × 10^−3^ (3.83 × 10^−4^)	NS
Leucine	4.40 × 10^−3^ (2.03 × 10^−3^)	3.94 × 10^−3^ (9.69 × 10^−4^)	NS
Methionine	9.26 × 10^−4^ (3.31 × 10^−4^)	8.07 × 10^−4^ (1.67 × 10^−4^)	NS
N-Methylhydantoin	3.15 × 10^−5^ (2.31 × 10^−5^)	3.97 × 10^−5^ (1.93 × 10^−5^)	NS
Orotate	6.04 × 10^−5^ (3.61 × 10^−5^)	7.25 × 10^−5^ (3.75 × 10^−5^)	NS
Phenylalanine	1.35 × 10^−3^ (7.01 × 10^−4^)	1.19 × 10^−3^ (1.97 × 10^−4^)	NS
Tyrosine	2.73 × 10^−3^ (1.23 × 10^−3^)	2.49 × 10^−3^ (4.87 × 10^−4^)	NS

Abbreviations: NGI, Children without gastrointestinal symptoms; GI, Children with gastrointestinal symptoms, NS, not significant.

**Table 3:** Key correlations in all the subjects studied among microbiology, calprotectin, and metabolomics data.

		Lact.	Akk.	Bifi.	Bact.	Prev.	Sutt.	Calpr.
Molecules altered only by GI	Formate	0.32	-	-	-	0.28	-	-
	Uridine	-	-	−0.26	0.30	-	-	−0.4
	Alanine	0.27	0.45	0.33	−0.46	-	−0.28	-
	Propionate	-	-	-	-	0.35	-	-
Molecules altered by GI and ADOS	Acetate	−0.23	-	−0.34	-	-	0.22	-
	Ethanol	−0.23	−0.28	-	-	-	0.28	-
	Isoleucine	0.37	-	0.51	−0.26	-	−0.38	-
	Leucine	0.41	0.27	0.54	−0.32	-	−0.41	-
	Methionine	-	0.23	0.33	-	-	-	-
	Orotate	-	-	−0.30	-	-	-	-
	Phenylalanine	0.32	-	0.46	-	-	−0.34	-
	Tyrosine	-	-	0.31	-	-	−0.32	-
Molecules altered only by ADOS	Aspartate	-	-	-	-	-	−0.44	-
	N-Methylhydantoin	-	-	-	-	−0.26	-	-
	1,3-Dihydroxyacetone	-	-	−0.23	-	-	-	-
	Fucose	-	-	-	0.26	-	0.31	−0.44
Metabolomics PC 1		**0.27**	**-**	**0.46**	**-**	**-**	**−0.35**	-

**Table 4:** Characteristics of the subjects in the whole sample and in the three severity subgroups.

	Whole Sample	Low-ADOS	Moderate-ADOS	High-ADOS
n	80	6	42	32
NGI/GI	52/28	4/2	29/13	19/13
Females/Males	14/66	2/4	9/33	3/29
Age (years)	4.14 ± 1.01	3.60 ± 1.01	4.29 ± 1.18	4.05 ± 0.97
BMI (Kg/m^2^)	16.00 ± 1.66	15.21 ± 1.61	15.81 ± 1.61	16.40 ± 1.69

Abbreviations: NGI, Children without gastrointestinal symptoms; GI, Children with gastrointestinal symptoms; BMI, Body Mass Index.

## Data Availability

The raw data supporting the conclusions of this article will be made available by the authors, without undue reservation.

## Supplementary Materials

The following are available online at https://www.mdpi.com/article/10.3390/metabo11100654/s1, Table S1: Concentration (mmol/L; median (IQR)) in feces of the molecules not significantly different between children without and with gastrointestinal symptoms, Table S2: Concentration (mmol/L; median (IQR)) in feces of the molecules not significantly different between children with high and low ADOS scores, Table S3: Correlation coefficients describing the rotation of the PCA space reported in Figure 1 and Figure 2 with respect to the original variable’s (molecules concentrations) system, Table S4: Copy numbers of selected bacteria in the feces of children. Values reported represent the median (IQR) of log10 concentration/g of feces, Table S5: Relative abundance of selected bacteria in the feces of children. Values reported represent the median (IQR) of the difference log_10_(concentration of targeted bacteria)—log_10_(concentration of total bacteria). * Significant differences (*p* < 0.05) between NGI and GI are reported in bold font, Table S6: Concentration of calprotectin in the feces of children (μg/g). Values reported represent the median (IQR) concentration/g of feces, Table S7: Correlation among relative abundance of bacteria in the feces of children, Table S8: *p*-values of two-way ANOVA (ADOS—gastrointestinal disease) models calculated on the relative abundance of the microorganisms, considering only children with low and high ADOS or all the children.

## Appendix A

### Appendix A.1. Metabolomics Analysis by ^1^H-NMR

A solution in D_2_O was created as a first step, containing 3-(trimethylsilyl)-propionic-2,2,3,3-d4 acid sodium salt (TSP) 10 mM and NaN_3_ 2 mM. The solution was set at pH 7.00 ± 0.02 by phosphate buffer 1 M. TSP served as NMR chemical-shift reference, while NaN_3_ avoided microbial proliferation.

In an Eppendorf tube, 80 mg of fecal samples were vortex mixed with 1 mL of deionized water and then centrifuged at 4 °C for 15 min at 18,630× *g*. The supernatant (0.7 mL was added to 0.1 mL of the above-described solution. After a further centrifugation step, ^1^H-NMR spectra were registered.

An AVANCE III spectrometer (Bruker, Milan, Italy), operating at a frequency of 600.13 MHz and equipped with the software Topspin 3.6, was employed to record the ^1^H-NMR spectra at 298 K. Presaturation was applied to suppress the residual signal from water, while broad signals from large molecules were reduced by a CPMG-filter, as outlined by Zhu et al. [55]. Each spectrum was acquired by summing up 256 transients, separated by 5 s relaxation delays, using 32 K data points over a 7184 Hz spectral window, with an acquisition time of 2.28 s.

Differences in water and solids content among samples were considered by probabilistic quotient normalization [56]. Spectra phase was manually adjusted in Topspin, while the subsequent adjustments were performed in R computational language by means of script developed in-house [52]. After the removal of the residual water signal, ^1^H-NMR spectra were baseline-corrected by means of peak detection, according to the “rolling ball” principle [57], implemented in the baseline R package [58]. Untargeted signals assignment was conducted by comparing their chemical shift and multiplicity with Chenomx software library (Chenomx Inc., Edmonton, AB, Canada, ver 8.3). For the purpose, each signal with a s/n ratio above 3 was subjected to scrutiny. Molecules’ quantification was performed by means of rectangular integration, considering one of the corresponding signals, free from interferences [59].

### Appendix A.2. Microbiota Analysis

Bifidobacteria and lactobacilli were quantified using *genus*-specific primers and conditions, as outlined by Matsuki et al. [60] and Štšepetova et al. [61], respectively. The Bacteroides/*Prevotella* group and *Prevotella genus* were determined using primers and protocols described by Bartosch et al. [62] and Larsen et al. [63], respectively. For *Sutterella genus* and *Akkermansia muciniphila* species, primers and PCR conditions were the same reported by Williams et al. [44] and Collado et al. [64], respectively. All samples were analyzed in duplicate and copy numbers values were obtained upon interpolation on standard curves built through serial 10-fold dilutions of bacterial DNA extracted from *Bifidobacterium breve*, *Lactobacillus brevis*, *Bacteroides fragilis*, *Prevotella pallens*, *Sutterella wadsworthensis*, and *A. muciniphila*. PCR was performed on optical-grade 96-well plates with Power SYBR GREEN PCRMaster Mix (Applied Biosystems, Foster City, CA, USA) using the Applied Biosystems 7500 real-time PCR instrument.
