# Supplementary material for: Are Fecal Metabolome and Microbiota Profiles Correlated with Autism Severity? A Cross-Sectional Study on ASD Preschoolers
Source: Metabolites. 2021 Sep 26;11(10):654. doi: 10.3390/metabo11100654 (PMC8539853; doi:10.3390/metabo11100654)
Supplement: Supplementary file 1 [file metabolites-11-00654-s001.zip › metabolites-1359495-supplementary.pdf]

Supplementary Materials

Appendix A

A.1. Metabolomics analysis by ^{1}H-NMR

A solution in D_2O was created as a first step, containing 3-(trimethylsilyl)-propionic-2,2,3,3-d4 acid sodium salt (TSP) 10 mM and NaN_3 2 mM. The solution was set at pH 7.00±0.02 by phosphate buffer 1M. TSP served as NMR chemical-shift reference, while NaN_3 avoided microbial proliferation.

In an Eppendorf tube, 80 mg of fecal samples were vortex mixed with 1 ml of deionized water and then centrifuged at 4°C for 15 min at 18630 g. The supernatant (0.7 mL was added to 0.1 mL of the above-described solution. After a further centrifugation step, ^{1}H-NMR spectra were registered.

An AVANCE III spectrometer (Bruker, Milan, Italy), operating at a frequency of 600.13 MHz and equipped with the software Topspin 3.6, was employed to record the ^{1}H-NMR spectra at 298 K. Presaturation was applied to suppress the residual signal from water, while broad signals from large molecules were reduced by a CPMG-filter, set as outlined by Zhu et al. (Laghi et al. 2018). Each spectrum was acquired by summing up 256 transients, separated by 5s relaxation delays, using 32 K data points over a 7184 Hz spectral window, with an acquisition time of 2.28s.

Differences in water and solids content among samples were considered by probabilistic quotient normalization (Dieterle et al. 2006). Spectra phase was manually adjusted in Topspin, while the subsequent adjustments were performed in R computational language by means of script developed in-house (R Development Core Team 3.0.1. 2013). After the removal of the residual water signal, ^{1}H-NMR spectra were baseline-corrected by means of peak detection, according to the “rolling ball” principle (Kneen and Annegarn 1996), implemented in the baseline R package (Liland et al. 2010). The signals were assigned by comparing their chemical shift and multiplicity with Chenomx software library (Chenomx Inc., Canada, ver 8.3). Molecules quantification was performed by means of rectangular integration, considering one of the corresponding signals, free from interferences (Foschi et al. 2018).

A.2. Microbiota analysis

In the microbiota analysis, we have chosen to investigate the bacterial species/genera most often described as altered in autism. In the text, it is identified simply with the term "microbiota" to improve readability.

Bifidobacteria and lactobacilli were quantified using *genus*-specific primers and conditions as Matsuki *et al.* (Matsuki et al. 2004) and Štšepetova *et al.* (Štšepetova et al. 2011), respectively. *Bacteroides*/*Prevotella* group and *Prevotella genus* were determined using primers and protocols described by Bartosch *et al.* (Bartosch et al. 2004) and Larsen *et al.* (Larsen et al. 2010), respectively. For *Sutterella genus* and *A. muciniphila* species, primers and PCR conditions were the same reported by Williams *et al.* (Williams et al. 2012) and Collado *et al.* (Collado et al. 2007), respectively. All samples were analyzed in duplicate and copy numbers values were obtained upon interpolation on standard curves built through serial 10-fold dilutions of bacterial DNA extracted from *Bifidobacterium breve, Lactobacillus brevis*, *Bacteroides fragilis*, *Prevotella pallens, Sutterella wadsworthensis* and *A. muciniphila*. PCR was performed on optical-grade 96-well plates with Power SYBR GREEN PCRMaster Mix (Applied Biosystems, Foster City, CA, USA) using the Applied Biosystems 7500 Real-Time PCR instrument.

Results: supplementary Tables

Table S1: Concentration (mmol/L; median (IQR)) in feces of the molecules not significantly different between children without and with gastrointestinal symptoms.

	NGI	GI	P-value
1,3-Dihydroxyacetone	$1.54x10^{-04}$ ($1.90x10^{-04}$)	$1.76x10^{-04}$ ($1.64x10^{-04}$)	0.21
2-Oxocaproate	$9.27x10^{-05}$ ($4.89x10^{-05}$)	$1.01x10^{-04}$ ($7.65x10^{-05}$)	0.97
2-Oxoisocaproate	$1.20x10^{-04}$ ($1.06x10^{-04}$)	$1.09x10^{-04}$ ($7.94x10^{-05}$)	0.48
3-Hydroxyphenylacetate	$2.14x10^{-04}$ ($1.65x10^{-04}$)	$2.77x10^{-04}$ ($2.33x10^{-04}$)	0.15
3-Methyl-2-oxovalerate	$1.85x10^{-04}$ ($1.41x10^{-04}$)	$1.60x10^{-04}$ ($1.91x10^{-04}$)	0.59
4-Aminobutyrate	$1.18x10^{-04}$ ($1.04x10^{-04}$)	$1.10x10^{-04}$ ($2.27x10^{-04}$)	0.75
4-Hydroxyphenylacetate	$9.44x10^{-04}$ ($7.39x10^{-04}$)	$1.04x10^{-03}$ ($3.81x10^{-04}$)	0.53
5-Aminopentanoate	$2.14x10^{-04}$ ($1.69x10^{-04}$)	$2.36x10^{-04}$ ($2.21x10^{-04}$)	0.81
Acetoacetate	$2.85x10^{-04}$ ($3.05x10^{-04}$)	$4.07x10^{-04}$ ($2.44x10^{-04}$)	0.99
Arabinose	$1.28x10^{-03}$ ($7.78x10^{-04}$)	$1.16x10^{-03}$ ($7.25x10^{-04}$)	0.13
Aspartate	$1.72x10^{-04}$ ($2.50x10^{-04}$)	$1.98x10^{-04}$ ($1.96x10^{-04}$)	0.37
β-Alanine	$1.42x10^{-02}$ ($1.83x10^{-02}$)	$1.31x10^{-02}$ ($2.19x10^{-02}$)	0.89
Butyrate	$1.07x10^{-04}$ ($4.98x10^{-05}$)	$9.96x10^{-05}$ ($4.29x10^{-05}$)	0.39
Creatine	$1.75x10^{-05}$ ($1.17x10^{-05}$)	$2.34x10^{-05}$ ($2.13x10^{-05}$)	0.39
Dimethylamine	$1.06x10^{-04}$ ($7.17x10^{-05}$)	$1.26x10^{-04}$ ($7.27x10^{-05}$)	0.69
Fucose	$3.54x10^{-04}$ ($3.82x10^{-04}$)	$3.66x10^{-04}$ ($3.57x10^{-04}$)	0.08
Fumarate	$1.47x10^{-04}$ ($8.37x10^{-05}$)	$1.83x10^{-04}$ ($1.04x10^{-04}$)	0.99
Galactose	$4.13x10^{-03}$ ($4.52x10^{-03}$)	$3.84x10^{-03}$ ($5.13x10^{-03}$)	0.14
Glucose	$4.06x10^{-03}$ ($2.37x10^{-03}$)	$3.47x10^{-03}$ ($1.48x10^{-03}$)	0.77
Glutamate	$2.85x10^{-04}$ ($4.27x10^{-04}$)	$7.93x10^{-04}$ ($5.93x10^{-03}$)	0.14
Glycerol	$1.84x10^{-03}$ ($1.03x10^{-03}$)	$1.56x10^{-03}$ ($8.01x10^{-04}$)	0.07
Glycine	$2.08x10^{-04}$ ($1.32x10^{-04}$)	$2.44x10^{-04}$ ($9.10x10^{-05}$)	0.37
Hypoxanthine	$5.72x10^{-03}$ ($3.70x10^{-03}$)	$6.54x10^{-03}$ ($4.47x10^{-03}$)	0.06
Isobutyrate	$1.30x10^{-03}$ ($9.59x10^{-04}$)	$1.19x10^{-03}$ ($1.09x10^{-03}$)	0.07

Isovalerate	$2.66x10^{-03}$ ($3.16x10^{-03}$)	$2.32x10^{-03}$ ($3.39x10^{-03}$)	0.99
Malonate	$2.61x10^{-04}$ ($2.00x10^{-04}$)	$4.21x10^{-04}$ ($4.75x10^{-04}$)	0.92
Methanol	$1.52x10^{-04}$ ($1.32x10^{-04}$)	$1.71x10^{-04}$ ($1.10x10^{-04}$)	0.06
Methylamine	$4.78x10^{-05}$ ($4.63x10^{-05}$)	$3.48x10^{-05}$ ($3.75x10^{-05}$)	0.12
N,N-Dimethylglycine	$1.73x10^{-04}$ ($1.13x10^{-04}$)	$2.18x10^{-04}$ ($8.63x10^{-05}$)	0.36
Nicotinate	$3.35x10^{-05}$ ($2.87x10^{-05}$)	$3.47x10^{-05}$ ($2.67x10^{-05}$)	0.08
N-Methylhydantoin	$4.22x10^{-05}$ ($5.72x10^{-05}$)	$5.42x10^{-05}$ ($1.10x10^{-04}$)	0.95
p-Cresol	$9.38x10^{-04}$ ($1.06x10^{-03}$)	$9.68x10^{-04}$ ($9.21x10^{-04}$)	0.19
Phenylacetate	$7.46x10^{-04}$ ($4.80x10^{-04}$)	$6.30x10^{-04}$ ($4.91x10^{-04}$)	0.76
Proline	$6.44x10^{-05}$ ($5.31x10^{-05}$)	$5.84x10^{-05}$ ($4.85x10^{-05}$)	0.53
Pyruvate	$1.24x10^{-05}$ ($1.11x10^{-05}$)	$1.34x10^{-05}$ ($1.14x10^{-05}$)	0.63
Sarcosine	$1.41x10^{-03}$ ($6.91x10^{-04}$)	$1.27x10^{-03}$ ($3.72x10^{-04}$)	0.47
Serine	$6.59x10^{-04}$ ($7.50x10^{-04}$)	$5.56x10^{-04}$ ($5.99x10^{-03}$)	0.65
Succinate	$1.96x10^{-03}$ ($8.02x10^{-04}$)	$1.76x10^{-03}$ ($6.13x10^{-04}$)	0.42
Threonine	$7.78x10^{-05}$ ($8.48x10^{-05}$)	$9.10x10^{-05}$ ($8.76x10^{-05}$)	0.34
TMA	$2.74x10^{-04}$ ($8.98x10^{-05}$)	$3.06x10^{-04}$ ($9.68x10^{-05}$)	0.40
Tryptophan	$6.68x10^{-05}$ ($8.95x10^{-05}$)	$8.83x10^{-05}$ ($8.30x10^{-05}$)	0.35
Tyramine	$7.82x10^{-04}$ ($3.68x10^{-04}$)	$7.39x10^{-04}$ ($3.44x10^{-04}$)	0.34
Uracil	$2.00x10^{-03}$ ($2.06x10^{-03}$)	$2.17x10^{-03}$ ($2.19x10^{-03}$)	0.47
Valerate	$2.52x10^{-03}$ ($1.10x10^{-03}$)	$2.10x10^{-03}$ ($7.86x10^{-04}$)	0.47
Valine	$2.76x10^{-04}$ ($1.62x10^{-04}$)	$2.84x10^{-04}$ ($1.12x10^{-04}$)	0.06
Xanthine	$1.34x10^{-04}$ ($1.92x10^{-04}$)	$1.28x10^{-04}$ ($1.40x10^{-04}$)	0.22
Xylose	$1.54x10^{-04}$ ($1.90x10^{-04}$)	$1.76x10^{-04}$ ($1.64x10^{-04}$)	0.95

Table S2: Concentration (mmol/L; median (IQR)) in feces of the molecules not significantly different between children with high and low ADOS scores.

	Low ADOS		Moderate ADOS		High ADOS		
	NGI	GI	NGI	GI	NGI	GI	P
Nicotinate	$1.25x10^{-4}$ ($4.01x10^{-5}$)	$2.04x10^{-4}$ ($3.63x10^{-5}$)	$1.81x10^{-4}$ ($7.63x10^{-5}$)	$2.31x10^{-4}$ ($4.64x10^{-5}$)	$1.53x10^{-4}$ ($1.77x10^{-4}$)	$1.64x10^{-4}$ ($1.18x10^{-4}$)	0.66
Formate	$1.03x10^{-4}$ ($1.85x10^{-5}$)	$1.47x10^{-4}$ ($4.77x10^{-5}$)	$1.32x10^{-4}$ ($5.39x10^{-5}$)	$1.73x10^{-4}$ ($8.84x10^{-5}$)	$1.01x10^{-4}$ ($5.61x10^{-5}$)	$1.34x10^{-4}$ ($5.03x10^{-5}$)	0.81
Hypoxanthine	$1.89x10^{-4}$ ($6.79x10^{-5}$)	$2.32x10^{-4}$ ($4.05x10^{-5}$)	$2.41x10^{-4}$ ($1.59x10^{-4}$)	$2.46x10^{-4}$ ($1.01x10^{-4}$)	$1.83x10^{-4}$ ($1.19x10^{-4}$)	$2.43x10^{-4}$ ($4.97x10^{-5}$)	0.95
Xanthine	$2.35x10^{-4}$ ($1.15x10^{-4}$)	$3.37x10^{-4}$ ($6.89x10^{-5}$)	$2.82x10^{-4}$ ($1.65x10^{-4}$)	$3.07x10^{-4}$ ($1.16x10^{-4}$)	$2.30x10^{-4}$ ($1.66x10^{-4}$)	$2.67x10^{-4}$ ($1.05x10^{-4}$)	0.94
Tryptophan	$3.20x10^{-4}$ ($8.86x10^{-5}$)	$2.82x10^{-4}$ ($3.47x10^{-5}$)	$2.72x10^{-4}$ ($8.84x10^{-5}$)	$2.72x10^{-4}$ ($6.96x10^{-5}$)	$2.67x10^{-4}$ ($6.57x10^{-5}$)	$3.28x10^{-4}$ ($7.94x10^{-5}$)	0.43
Phenylacetate	$6.08x10^{-4}$ ($3.82x10^{-4}$)	$1.37x10^{-3}$ ($1.83x10^{-4}$)	$1.10x10^{-3}$ ($1.12x10^{-3}$)	$8.38x10^{-4}$ ($5.17x10^{-4}$)	$8.71x10^{-4}$ ($5.31x10^{-4}$)	$1.04x10^{-3}$ ($2.66x10^{-3}$)	0.38
3-Hydroxyphenylacetate	$2.20x10^{-4}$ ($1.46x10^{-4}$)	$2.15x10^{-4}$ ($1.53x10^{-4}$)	$2.14x10^{-4}$ ($1.82x10^{-4}$)	$2.65x10^{-4}$ ($1.66x10^{-4}$)	$1.91x10^{-4}$ ($1.73x10^{-4}$)	$3.39x10^{-4}$ ($2.07x10^{-4}$)	0.74
Tyramine	$1.12x10^{-4}$ ($4.89x10^{-5}$)	$2.84x10^{-5}$ ($6.31x10^{-6}$)	$6.29x10^{-5}$ ($9.33x10^{-5}$)	$8.93x10^{-5}$ ($5.50x10^{-5}$)	$5.75x10^{-5}$ ($6.03x10^{-5}$)	$1.06x10^{-4}$ ($9.22x10^{-5}$)	0.70
4-Hydroxyphenylacetate	$1.16x10^{-4}$ ($3.25x10^{-5}$)	$5.84x10^{-5}$ ($4.51x10^{-6}$)	$1.16x10^{-4}$ ($1.15x10^{-4}$)	$1.19x10^{-4}$ ($9.23x10^{-5}$)	$1.30x10^{-4}$ ($1.04x10^{-4}$)	$9.91x10^{-5}$ ($3.48x10^{-4}$)	0.08
pCresol	$2.65x10^{-5}$ ($9.99x10^{-5}$)	$5.42x10^{-5}$ ($2.61x10^{-5}$)	$4.80x10^{-5}$ ($6.19x10^{-5}$)	$5.33x10^{-5}$ ($2.67x10^{-5}$)	$3.96x10^{-5}$ ($4.57x10^{-5}$)	$8.79x10^{-5}$ ($1.76x10^{-4}$)	0.53
Fumarate	$1.09x10^{-4}$ ($8.28x10^{-6}$)	$2.52x10^{-4}$ ($1.36x10^{-4}$)	$4.47x10^{-4}$ ($3.44x10^{-4}$)	$3.48x10^{-4}$ ($4.35x10^{-4}$)	$2.88x10^{-4}$ ($3.78x10^{-4}$)	$3.84x10^{-4}$ ($3.63x10^{-4}$)	0.05
Uridine	$5.74x10^{-5}$ ($6.63x10^{-5}$)	$7.28x10^{-5}$ ($1.91x10^{-5}$)	$4.56x10^{-5}$ ($3.91x10^{-5}$)	$8.13x10^{-5}$ ($5.78x10^{-5}$)	$4.46x10^{-5}$ ($3.10x10^{-5}$)	$5.30x10^{-5}$ ($5.11x10^{-5}$)	0.58
Uracil	$7.67x10^{-4}$ ($1.70x10^{-4}$)	$1.08x10^{-3}$ ($9.01x10^{-5}$)	$8.40x10^{-4}$ ($5.02x10^{-4}$)	$8.03x10^{-4}$ ($7.41x10^{-4}$)	$7.16x10^{-4}$ ($5.00x10^{-4}$)	$6.48x10^{-4}$ ($2.70x10^{-4}$)	0.33

Galactose	1.30x10^{-4} (4.52x10^{-5})	1.41x10^{-4} (2.47x10^{-6})	1.38x10^{-4} (7.28x10^{-5})	1.97x10^{-4} (1.02x10^{-4})	1.89x10^{-4} (1.07x10^{-4})	1.58x10^{-4} (1.19x10^{-4})	0.34
Xylose	2.00x10^{-4} (1.27x10^{-4})	1.11x10^{-4} (2.06x10^{-5})	1.15x10^{-4} (1.69x10^{-4})	1.83x10^{-4} (1.61x10^{-4})	1.38x10^{-4} (2.13x10^{-4})	1.25x10^{-4} (1.19x10^{-4})	0.85
Arabinose	2.47x10^{-4} (2.06x10^{-4})	2.64x10^{-4} (3.14x10^{-5})	2.94x10^{-4} (1.82x10^{-4})	4.72x10^{-4} (2.49x10^{-4})	2.76x10^{-4} (3.51x10^{-4})	4.07x10^{-4} (1.87x10^{-4})	0.56
Threonine	1.96x10^{-3} (2.38x10^{-4})	2.27x10^{-3} (3.26x10^{-4})	2.11x10^{-3} (8.36x10^{-4})	1.96x10^{-3} (5.61x10^{-4})	1.78x10^{-3} (8.57x10^{-4})	1.52x10^{-3} (6.86x10^{-4})	0.26
Serine	1.71x10^{-3} (4.65x10^{-4})	1.81x10^{-3} (5.95x10^{-5})	1.49x10^{-3} (5.66x10^{-4})	1.26x10^{-3} (1.95x10^{-4})	1.28x10^{-3} (8.00x10^{-4})	1.18x10^{-3} (4.43x10^{-4})	0.08
Glycerol	1.65x10^{-4} (3.74x10^{-5})	2.02x10^{-2} (1.99x10^{-2})	2.88x10^{-4} (4.22x10^{-4})	1.29x10^{-3} (6.97x10^{-3})	3.51x10^{-4} (5.02x10^{-4})	4.24x10^{-4} (6.06x10^{-4})	0.69
Glycine	2.00x10^{-3} (3.54x10^{-4})	2.51x10^{-3} (5.74x10^{-4})	1.94x10^{-3} (1.08x10^{-3})	1.68x10^{-3} (6.00x10^{-4})	1.66x10^{-3} (1.23x10^{-3})	1.23x10^{-3} (4.72x10^{-4})	0.32
Methanol	2.23x10^{-4} (7.71x10^{-5})	5.47x10^{-4} (1.79x10^{-4})	2.26x10^{-4} (2.03x10^{-4})	2.94x10^{-4} (4.87x10^{-4})	2.81x10^{-4} (4.02x10^{-4})	4.65x10^{4} (3.50x10^{-4})	0.62
Proline	7.35x10^{-4} (4.25x10^{-4})	1.03x10^{-3} (1.49x10^{-4})	7.51x10^{-4} (3.11x10^{-4})	6.93x10^{-4} (4.25x10^{-4})	7.31x10^{-4} (6.86x10^{-4})	4.61x10^{-4} (2.70x10^{-4})	0.22
Glucose	6.37x10^{-3} (5.78x10^{-3})	5.20x10^{-3} (3.05x10^{-3})	4.17x10^{-3} (4.55x10^{-3})	4.30x10^{-3} (7.48x10^{-3})	4.08x10^{-3} (3.74x10^{-3})	3.46x10^{-3} (3.73x10^{-3})	0.67
Malonate	5.49x10^{-3} (1.17x10^{-2})	1.70x10^{-3} (6.07x10^{-4})	2.12x10^{-3} (1.78x10^{-3})	1.81x10^{-3} (3.32x10^{-3})	3.41x10^{-3} (3.81x10^{-3})	2.68x10^{-3} (3.95x10^{-3})	0.89
Creatine	1.08x10^{-4} (1.42x10^{-5})	1.02x10^{-4} (1.51x10^{-5})	1.06x10^{-4} (5.32x10^{-5})	8.69x10^{-5} (3.48x10^{-5})	1.13x10^{-4} (4.85x10^{-5})	1.03x10^{-4} (6.46x10^{-5})	0.88
N.N-Dimethylglycine	7.23x10^{-5} (6.01x10^{-5})	1.06x10^{-4} (5.37x10^{-5})	4.88x10^{-5} (4.48x10^{-5})	3.38x10^{-5} (3.73x10^{-5})	3.81x10^{-5} (3.40x10^{-5})	3.46x10^{-5} (2.78x10^{-5})	0.10
TMA	5.13x10^{-5} (1.08x10^{-5})	1.18x10^{-4} (4.89x10^{-5})	8.38x10^{-5} (8.49x10^{-5})	7.43x10^{-5} (5.02x10^{-5})	7.50x10^{-5} (1.05x10^{-4})	1.23x10^{-4} (1.04x10^{-4})	0.43
Sarcosine	8.24x10^{-6} (2.18x10^{-6})	1.28x10^{-5} (1.74x10^{-6})	1.21x10^{-5} (1.11x10^{-5})	1.20x10^{-5} (1.38x10^{-5})	1.69x10^{-5} (1.36x10^{-5})	1.44x10^{-5} (6.81x10^{-6})	0.15
2-Oxocaproate	8.17x10^{-5} (2.21x10^{-5})	1.52x10^{-4} (4.10x10^{-5})	9.30x10^{-5} (4.23x10^{-5})	9.36x10^{-5} (7.98x10^{-5})	9.91x10^{-5} (6.37x10^{-5})	1.02x10^{-4} (8.50x10^{-5})	0.76
Dimethylamine	1.62x10^{-5} (3.54x10^{-6})	2.76x10^{-5} (1.24x10^{-5})	1.79x10^{-5} (1.28x10^{-5})	2.34x10^{-5} (1.28x10^{-5})	1.71x10^{-5} (1.28x10^{-5})	2.61x10^{-5} (2.40x10^{-5})	0.94
2-Oxoisocaproate	1.20x10^{-4} (5.11x10^{-5})	1.73x10^{-4} (2.12x10^{-5})	1.27x10^{-4} (1.16x10^{-4})	1.24x10^{-4} (9.51x10^{-5})	1.09x10^{-4} (7.35x10^{-5})	9.20x10^{-5} (5.39x10^{-5})	0.57
Methylamine	9.13x10^{-5} (1.74x10^{-4})	1.99x10^{-4} (2.79x10^{-5})	1.54x10^{-4} (8.42x10^{-5})	1.71x10^{-4} (9.43x10^{-5})	1.74x10^{-4} (1.63x10^{-4})	1.75x10^{-4} (1.24x10^{-4})	0.85
β-Alanine	1.68x10^{-4} (1.90x10^{-4})	1.09x10^{-4} (3.27x10^{-6})	1.55x10^{-4} (1.43x10^{-4})	2.36x10^{-4} (5.08x10^{-4})	2.13x10^{-4} (4.34x10^{-4})	1.74x10^{-4} (1.44x10^{-4})	0.40
Succinate	1.17x10^{-3} (1.15x10^{-2})	4.31x10^{-4} (3.35x10^{-5})	4.84x10^{-4} (4.51x10^{-4})	5.41x10^{-4} (4.90x10^{-3})	8.17x10^{-4} (1.74x10^{-3})	6.08x10^{-4} (9.48x10^{-3})	0.81
Pyruvate	6.06x10^{-5} (1.68x10^{-5})	7.82x10^{-5} (1.95x10^{-5})	6.63x10^{-5} (5.26x10^{-5})	5.74x10^{-5} (6.05x10^{-5})	7.11x10^{-5} (5.46x10^{-5})	5.81x10^{-5} (3.91x10^{-5})	0.77
Glutamate	3.56x10^{-3} (8.90x10^{-4})	4.71x10^{-3} (5.27x10^{-4})	4.21x10^{-3} (1.83x10^{-3})	3.49x10^{-3} (2.59x10^{-3})	3.88x10^{-3} (2.58x10^{-3})	2.94x10^{-3} (1.39x10^{-3})	0.46
4-Aminobutyrate	2.00x10^{-4} (5.29x10^{-5})	1.75x10^{-4} (2.38x10^{-6})	1.72x10^{-4} (1.04x10^{-4})	2.15x10^{-4} (1.69x10^{-4})	2.32x10^{-4} (2.90x10^{-4})	1.79x10^{-4} (7.27x10^{-5})	0.53
Acetoacetate	1.72x10^{-4} (8.77x10^{-5})	2.11x10^{-4} (6.13x10^{-5})	2.16x10^{-4} (2.12x10^{-4})	1.41x10^{-4} (1.98x10^{-4})	2.28x10^{-4} (1.47x10^{-4})	2.73x10^{-4} (2.29x10^{-4})	0.52
5-Aminopentanoate	7.25x10^{-4} (8.60x10^{-4})	6.38x10^{-4} (8.48x10^{-5})	8.88x10^{-4} (5.29x10^{-4})	1.11x10^{-3} (4.01x10^{-4})	1.04x10^{-3} (9.65x10^{-4})	9.44x10^{-4} (3.62x10^{-4})	0.31
Alanine	5.74x10^{-3} (2.21x10^{-3})	6.47x10^{-3} (8.99x10^{-5})	6.61x10^{-3} (1.92x10^{-0})	4.55x10^{-3} (2.07x10^{-3})	5.88x10^{-3} (1.68x10^{-3})	4.87x10^{-3} (1.40x10^{-3})	0.46
3-Methyl-2-oxovalerate	1.15x10^{-4} (1.11x10^{-4})	1.47x10^{-4} (4.68x10^{-5})	1.85x10^{-4} (7.91x10^{-5})	1.73x10^{-4} (3.25x10^{-4})	1.95x10^{-4} (1.92x10^{-4})	1.52x10^{-4} (9.78x10^{-5})	0.47
Isobutyrate	4.38x10^{-3} (1.59x10^{-3})	7.86x10^{-3} (1.01x10^{-3})	7.18x10^{-3} (3.19x10^{-3})	5.59x10^{-3} (1.98x10^{-3})	5.20x10^{-3} (2.96x10^{-3})	8.45x10^{-3} (7.15x10^{-3})	0.42
Propionate	1.49x10^{-2} (4.80x10^{-3})	2.53x10^{-2} (2.33x10^{-3})	2.24x10^{-2} (9.38x10^{-3})	1.79x10^{-2} (5.43x10^{-3})	1.65x10^{-2} (1.06x10^{-2})	2.92x10^{-2} (2.03x10^{-2})	0.52
Valine	2.60x10^{-3} (6.83x10^{-4})	2.91x10^{-3} (1.84x10^{-7})	2.56x10^{-3} (1.04x10^{-3})	2.18x10^{-3} (5.23x10^{-4})	2.42x10^{-3} (1.43x10^{-3})	2.06x10^{-3} (5.65x10^{-4})	0.06
Isovalerate	8.88x10^{-4} (4.04x10^{-4})	1.85x10^{-3} (2.61x10^{-4})	1.56x10^{-3} (1.55x10^{-3})	9.69x10^{-4} (7.33x10^{-4})	1.28x10^{-3} (7.30x10^{-4})	1.25x10^{-3} (3.14x10^{-3})	0.40
Butyrate	6.01x10^{-3} (5.17x10^{-3})	1.68x10^{-2} (7.74x10^{-3})	1.49x10^{-2} (1.28x10^{-2})	1.05x10^{-2} (4.14x10^{-3})	1.68x10^{-2} (2.37x10^{-2})	2.93x10^{-2} (2.04x10^{-2})	0.07
Valerate	8.53x10^{-4} (9.11x10^{-4})	2.47x10^{-3} (7.18x10^{-4})	2.44x10^{-3} (1.62x10^{-3})	1.67x10^{-3} (1.82x10^{-3})	1.50x10^{-3} (1.73x10^{-3})	2.69x10^{-3} (3.55x10^{-3})	0.14

Table S3: Correlation coefficients describing the rotation of the PCA space reported in figure 1 and 2 with respect to the original variable's (molecules concentrations) system.

	PC 1	PC 2	PC 3
1,3-Dihydroxyacetone	$-1.07x10^{-1}$	$-7.82x10^{-1}$	$4.52x10^{-1}$
Acetate	$-3.02x10^{-1}$	$-2.82x10^{-1}$	$-4.51x10^{-1}$
Aspartate	$3.25x10^{-1}$	$-1.17x10^{-1}$	$1.45x10^{-1}$
Ethanol	$-1.45x10^{-1}$	$-1.39x10^{-1}$	$3.86x10^{-1}$
Fucose	$-5.26x10^{-2}$	$-6.51x10^{-2}$	$2.91x10^{-2}$
Isoleucine	$3.90x10^{-1}$	$-1.14x10^{-1}$	$-5.93x10^{-2}$
Leucine	$4.03x10^{-1}$	$-1.04x10^{-1}$	$-6.99x10^{-2}$
Methionine	$4.08x10^{-1}$	$-9.83x10^{-2}$	$-7.20x10^{-2}$
N-Methylhydantoin	$-3.52x10^{-2}$	$-4.43x10^{-2}$	$1.98x10^{-1}$
Orotate	$-5.14x10^{-2}$	$-4.63x10^{-1}$	$-6.04x10^{-1}$
Phenylalanine	$3.85x10^{-1}$	$2.84x10^{-3}$	$-7.58x10^{-3}$
Tyrosine	$3.67x10^{-1}$	$-1.47x10^{-1}$	$-4.89x10^{-2}$

Table S4: Copy numbers of selected bacteria in the feces of children.

	**Total subjects**	**NGI**	**GI**	**Low ADOS**		**Moderate ADOS**		**High ADOS**	
				**NGI**	**GI**	**NGI**	**GI**	**NGI**	**GI**
Total bacteria	10.25 (0.41)	10.21 (0.34)	10.37 (0.31)	10.21 (0.09)	10.38 (0.23)	10.26 (0.31)	10.21 (0.35)	10.19 (0.56)	10.43 (0.21)
Lactobacilli	4.92 (1.25)	4.88 (1.14)	4.94 (1.32)	5.10 (0.51)	5.28 (0.34)	4.72 (1.11)	4.90 (1.44)	4.86 (1.22)	4.99 (1.28)
A. muciniphila	6.79 (3.98)	6.99 (3.88)	6.14 (4.16)	6.89 (2.19)	8.13 (0.07)	7.25 (2.29)	5.70 (4.10)	5.81 (4.53)	6.00 (4.34)
Bifidobacteria	8.55 (0.53)	8.56 (0.50)	8.53 (0.62)	8.36 (0.97)	8.22 (0.14)	8.55 (0.40)	8.51 (0.36)	8.57 (0.52)	8.58 (0.79)
Bacteroides	9.09 (0.67)	9.05 (0.58)	9.33 (0.57)	9.19 (0.18)	8.87 (0.48)	8.97 (0.54)	9.23 (0.61)	9.04 (0.80)	9.45 (0.55)
Prevotella	4.06 (1.17)	4.11 (1.07)	3.91 (1.55)	3.85 (1.02)	5.78 (1.92)	4.15 (2.83)	3.86 (0.64)	4.04 (0.84)	4.00 (2.40)
Sutterella	6.69 (1.39)	6.67 (1.50)	6.93 (1.21)	6.68 (0.31)	5.01 (1.50)	6.57 (1.19)	6.22 (1.21)	6.67 (2.20)	7.37 (0.47)

Values reported represent the median (IQR) of log10 concentration/g of feces

Table S5: Relative abundance of selected bacteria in the feces of children.

	**Total subjects**	**NGI***	**GI***	**Low ADOS**		**Moderate ADOS**		**High ADOS**	
				**NGI**	**GI**	**NGI**	**GI**	**NGI**	**GI**
Lactobacilli	-5.32 (1.34)	-5.38 (1.14)	-5.20 (1.50)	-5.10 (0.34)	-5.10 (0.12)	-5.53 (1.28)	-5.25 (1.97)	-5.37 (1.01)	-5.18 (1.37)
A. muciniphila	-3.52 (4.01)	-3.49 (3.62)	-4.00 (4.33)	-3.37 (2.29)	-2.25 (0.30)	-3.00 (2.58)	-4.16 (4.04)	-3.81 (4.24)	-4.63 (4.53)
Bifidobacteria	-1.75 (0.35)	**-1.70 (0.37)**	**-1.86 (0.39)**	-1.84 (0.79)	-2.16 (0.37)	-1.70 (0.35)	-1.79 (0.32)	-1.64 (0.40)	-1.96 (0.64)

Bacteroides	-1.15 (0.49)	-1.20 (0.46)	-1.01 (0.50)	-1.02 (0.21)	-1.52 (0.26)	-1.23 (0.44)	-0.98 (0.42)	-1.08 (0.50)	-1.01 (0.48)
Prevotella	-6.12 (1.13)	-6.06 (1.14)	-6.24 (1.37)	-6.32 (1.15)	-4.61 (1.70)	-5.97 (2.85)	-6.36 (0.84)	-6.17 (0.97)	-6.23 (2.26)
Sutterella	-3.53 (1.04)	-3.53 (1.04)	-3.48 (0.97)	-3.49 (0.44)	-5.38 (1.27)	-3.52 (1.11)	-3.82 (1.14)	-3.74 (1.83)	-3.13 (0.66)

Values reported represent the median (IQR) of the difference $\log_{10}$(concentration of targeted bacteria) - $\log_{10}$(concentration of total bacteria)

*Significant differences ($p < 0.05$) between NGI and GI are reported in bold font

Table S6: Concentration of calprotectin in the feces of children (µg/g)

	Total subjects	NGI	GI	Low ADOS		Moderate ADOS		High ADOS	
				**NGI**	**GI**	**NGI**	**GI**	**NGI**	**GI**
Calprotectin	77.12 (135.69)	79.27 (131.15)	69.50 (131.21)	33.70 (22.88)	78.64 (16.34)	80.91 (137.97)	93.56 (137.99)	86.75 (139.5)	66.47 (56.67)

Values reported represent the median (IQR) concentration/g of feces

Table S7: Correlation among relative abundance of bacteria in the feces of children.

	Sutterella	*Prevotella*	*Bacteroides*	Bifidobacteria	*A. muciniphila*
Lactobacilli	-0.36	0.38	-	0.38	-
A. muciniphila	-	-	-0.33	-	
Bifidobacteria	-0.37	-	-0.35		
Bacteroides	0.54	-			
Prevotella	-				

Table S8: P-values of two-way ANOVA (ADOS – gastrointestinal disease) models calculated on the relative abundance of the microorganisms, considering only children with Low and High ADOS or all the children.

	Low and High ADOS children		All the children	
	ADOS score	Gastrointestinal disease	ADOS score	Gastrointestinal disease
Lactobacilli	0.539	0.485	0.852	0.544
A. muciniphila	0.342	0.587	0.365	0.351
Bifidobacteria	0.096	0.072	0.107	0.071
Bacteroides	0.822	0.410	0.934	0.845
Prevotella	0.736	0.056	0.384	0.498
Sutterella	0.391	**0.036**	0.473	0.264

Abbreviations: ADOS: NGI, Children without gastrointestinal symptoms; GI, Children with gastrointestinal symptoms
